# Implantable Biosensors for Vascular Diseases: Directions for the Next Generation of Active Diagnostic and Therapeutic Medical Device Technologies

**DOI:** 10.3390/bios15030147

**Published:** 2025-02-25

**Authors:** Ali Mana Alyami, Mahmut Talha Kirimi, Steven L. Neale, John R. Mercer

**Affiliations:** 1 BHF Cardiovascular Research Centre, University of Glasgow, Glasgow G12 8TA, UK; a.alyami.1@research.gla.ac.uk (A.M.A.); mahmuttalha.kirimi@glasgow.ac.uk (M.T.K.); 2 James Watt South Building, College of Science and Engineering, University of Glasgow, Glasgow G12 8QQ, UK; steven.neale@glasgow.ac.uk

**Keywords:** coronary artery disease, atherosclerosis, in-stent restenosis, chronic kidney disease, haemodialysis, arteriovenous fistula and graft, biosensors, smart implantable medical devices, and wireless power and data transfer

## Abstract

Cardiovascular disease remains the leading cause of morbidity and mortality worldwide. Key challenges such as atherosclerosis, in-stent restenosis, and maintaining arteriovenous access, pose urgent problems for effective treatments for both coronary artery disease and chronic kidney disease. The next generation of active implantables will offer innovative solutions and research opportunities to reduce the economic and human cost of disease. Current treatments rely on vascular stents or synthetic implantable grafts to treat vessels when they block such as through in-stent restenosis and haemodialysis graft failure. This is often driven by vascular cell overgrowth termed neointimal hyperplasia, often in response to inflammation and injury. The integration of biosensors into existing approved implants will bring a revolution in cardiovascular devices and into a promising new era. Biosensors that allow real-time vascular monitoring will provide early detection and warning of pathological cell growth. This will enable proactive wireless treatment outside of the traditional hospital settings. Ongoing research focuses on the development of self-reporting smart cardiovascular devices, which have shown promising results using a combination of virtual in silico modelling, bench testing, and preclinical in vivo testing. This innovative approach holds the key to a new generation of wireless data solutions and wireless powered implants to enhance patient outcomes and alleviate the burden on global healthcare budgets.

## 1. Introduction

### 1.1. Neointimal Hyperplasia

Neointimal hyperplasia (NIH) and thrombosis are the biological processes that drive the majority of vascular implant failures. NIH is a wound response that promotes blood clot formation in vessels in response to significant luminal injury. Vascular treatments, including percutaneous coronary intervention (PCI), coronary artery bypass grafting (CABG), arteriovenous grafting (AVG), and arteriovenous fistula (AVF) establishment, are major contributors in the development of NIH and thrombosis, despite their substantial benefits. NIH is classified into two types: (1) arterial NIH caused by mechanical trauma during PCI, and (2) venous graft NIH resulting from CABG, AVG and/or AVF formation in haemodialysis patients [1,2].

**Figure 1:**
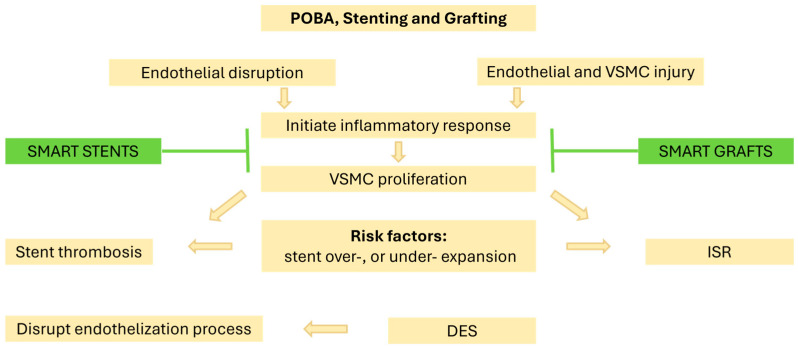
Illustration of the impact imposed by PCI, grafting, and DES. PCI and grafting may cause procedure-related injury to the vascular wall. Such injury can initiate an inflammatory cascade, which causes VSMCs to proliferate and migrate at the intimal layer. This leads to the development of NIH and/or ISR. Current available DESs effectively reduce the risk of restenosis; however, they can obstruct the endothelial healing process, increasing the risk of stent thrombosis.

The development of NIH, as depicted in Figure 1, can initially be due to injury of the delicate inner endothelium vessel layer during the implantation of prosthetic devices. For example, the overexpansion of deployed stent [3]. Endothelium damage induces a complex inflammatory response, which triggers the migration and proliferation of vascular smooth muscle cells (VSMCs) at the tunica intima. Cell accumulation in the tunica intima layer thickens the arterial wall and leads to a loss of patency [4]. Later, affected arterial sites are highly susceptible to the build-up of lipoprotein, which accelerates the progression of atherosclerosis [5].

Many research studies have proven that the stent type and shape can induce an inflammatory response [6]. Therefore, stents have been through several generations to enhance clinical outcomes and mitigate side effects after deployment. Bare metal stents (BMS) are associated with a high risk of restenosis and the need for repeat revascularization, whereas drug eluting stents (DESs) have shown more favourable outcomes in patients by reducing the risk of in-stent restenosis (ISR) [7,8]. On the other hand, vascular access, used for haemodialysis treatment in chronic kidney disease (CKD) patients, are at a high risk of failure too due to NIH [9,10]. The failure of these accesses cannot be corrected and rather, patients will have to undergo another procedure to establish another access.

### 1.2. Unresolved Challenges in the Treatment of Neointimal Hyperplasia

Plain old balloon angioplasty (POBA) was first performed in the 1970s as a revolutionary alternative to coronary artery bypass graft surgery (CABG). However, coronary POBA was associated with a high risk of NIH, sudden vessel closure, and elastic recoil. As a result, many patients underwent open-heart surgery due to these abrupt issues [11,12]. In the mid-1980s, a self-expandable stent known as Wallstent™ attempted to address these issues. However, by 1991, Wallstent™ stents were withdrawn from the market due to technical challenges with their delivery system. This remains an ongoing challenge when targeting culprit lesions in difficult-to-access vessel segments [13]. Johnson & Johnson^TM^ developed the first bare metal stent (BMS) in the 1980s, which became the first FDA-approved metal stent over balloon. The use of BMS significantly decreased the incidence of vascular recoil and restenosis to 17–44% compared to POBA [12,13]. However, BMS did not fully resolve the issue, as it could still induce NIH in up to 30% of patients [14]. This often required urgent and costly repeat interventions of PCI to re-open the re-stenotic BMS.

The drawbacks of BMS paved the way for the development of drug eluting stents (DESs). Stents coated with an antiproliferative drug in the early 2000s revolutionised the field of PCI. DESs have evolved through multiple generations. First-generation DES strut structures were fabricated of stainless steel and were coated with either the mTOR inhibitor rapamycin (Sirolimus) or the anti-tubulin paclitaxel (Taxol). Second-generation DES used cobalt–chromium struts and memory metals such as nitinol, which were coated with everolimus or zotarolimus. This generation benefited from reduced strut thickness, which improved flexibility, biocompatibility, and better eluting characteristics [15]. The most common advantage of DESs is their ability to lower the incidence of lumen re-blockage to approximately 10% [16,17]. Despite this benefit, while DES can potentially reduce the risk of early restenosis and NIH, clinical trials have reported manifestations of late (>30 days) and very late (>12 months) stent thrombosis. This is owed to the antiproliferative drugs in DES, whose drug release kinetics can mismatch with the evolving pathology while slowing down the process of stent endothelization [14]. Once the systemic antiplatelets course therapy is completed, the uncovered parts of the strut can trigger an inflammatory response and lead to late stent thrombosis, which remains a challenge. Third-generation DESs were the bioabsorbable scaffold made from dissolvable polylactic acid (PLA). These emerged to avoid the lifetime implantation of conventional stents due to their ability to completely degrade within the body tissue. Randomised trials such as ABSORB II, ABSORB III, ABSORB China, and ABSORB, Japan, concluded that bioresorbable stents were associated with high rates of intrastent strut scaffold collapse that promoted thrombosis, and the trials were stopped [17].

In contrast, stent technologies continue to be used in combination with polytetrafluoroethylene (PTFE) AVGs. The creation of AVGs is susceptible to failure as a result of venous-induced NIH followed by thrombosis. Currently, graft failure cannot be easily treated; however, the placement of another intragraft inside the original within or across the culprit lesion is now common. The Gore Viabhan^TM^ stent graft endoprosthesis technology has been a highly effective solution. The burden of graft failures and their impact on patients, healthcare, and cost are discussed in more detail in Section 3 but remain the driver for technological innovation with the HealthTech and MedTech sectors.

To combat these weaknesses and challenges caused by conventional stents and grafts, cardiovascular (CVD) and peripheral vascular disease (PVD) researchers are working towards the development of the next generation of smart stents and grafts. These innovative devices are designed to detect cellular growth which promotes thrombosis (blood clots) at the very earliest presymptomatic stage, thereby “buying time”. More than just diagnostic, they provide a remote early warning system. By embedding novel biosensors, which can detect vascular complications, they offer proactive wireless treatment, eliminating the need for hospitalisation. This literature review reflects the primary aim of our project towards the development of innovative smart stents and grafts, capable of conducting diagnosis and applying therapy wirelessly. To support our goal, we evaluated the existing literature on CVD, the failure of AVF and AVG, for smart implantable devices.

For sure, the challenges in this field for the integration of the technology into and onto existing implants are huge. The miniaturisation of the associated electronics, software, firmware for wireless data, and power transfer are major technical challenges. The regulatory framework and approvals for non-predicate devices to ensure reimbursement strategies are also key. Yet, biosensors on implants offer the lofty goal of detecting and relaying previously unobtainable clinical actionable data. However, the data still need to be integrated as part of a normal clinical workflow to be effective. Nevertheless, the economic benefits are huge; the stent market is estimated at USD 12.73 Bn, and the graft markets at USD 5.4 Bn. The reduction in patients needing to attend hospital appointments through “at-home” surveillance reduces clinician and patient stress. The offers of a remote therapy are highly sought after. The technology is coming, and when integrated with artificial intelligence for predicted mean time before failure (MTBF) of the implant, it will revolutionise the stent graft market, especially for those companies willing to act as early adopters. To further our understanding of current advancements, challenges, and gaps in the field, this review serves as a guide the towards the development of the next generation of active diagnostic and therapeutic medical device technologies.

## 2. Cardiovascular Disease

CVD is an umbrella term used to describe various conditions affecting the heart or blood vessels. CVD continues to be the leading cause of both mortality and morbidity in the world. The World Health Organisation (WHO) reports that the deaths from CVD in 2019 were estimated at 17.9 million. This represents 32% of all deaths around the world. According to the WHO, 85% of all CVD deaths are due to stroke and heart attack [18]. The prevalence of CVD has significantly doubled from 271 million in 1990 to 523 million in 2019 worldwide. The burden of CVD continues to increase globally and mostly affects economically disadvantaged populations [19]. The British Heart Foundation (BHF) states that CVD predominantly affects males, with 4 million cases, as opposed to 3.6 million cases in females. The number of deaths within the United Kingdom (UK) resulting from CVD is estimated at more than 170,000 cases, constituting 27% of all deaths in the country [20].

The predominate types of CVD includes coronary artery disease (CAD), peripheral vascular disease, aortic disease, and cerebrovascular disease [21]. Each condition has its own specific pathophysiology, risk factors and treatment approach. Coronary and peripheral arteries diseases are of major interest, from both the human and economic perspective.

Key CVD risk factors are unmodifiable such as genetic disorders, gender, age and familial hypercholesterolaemia. However, many risk factors including diabetes, hypertension, smoking, alcohol abuse, hypercholesterolemia, and unhealthy diet that can be controlled [22,23]. Prevention and management of such risk factors require lifestyle modifications and compliance to medications. For example, physical activity, balanced healthy diet, compliance with medications and cigarettes cessation [24].

The impact of CVD extends beyond the overall public health, reaching to the healthcare budgets. That being said, the economic cost of CVD as noted is substantial, adding a significant cost burden for both individuals and healthcare services around the world. The American Heart Association (AHA) states that in the United States (US), the direct and indirect cost of CVD was estimated at USD 407.3 billion in 2018–2019. Direct costs such as the hospitalisations and medications of CVD has significantly increased from USD 103.5 to USD 251.4 billion between 1996 and 2019, respectively. However, in the fiscal year 2018–2019, hospital inpatients resulted in the highest direct expenditure, totalling USD 111.4 billion [25]. In addition, the socioeconomic status of people plays a crucial role in the development of CVD. Low socioeconomic status is linked to a high incidence of CVD [26].

### 2.1. Coronary Artery Disease

#### 2.1.1. Anatomy

The coronary arteries (CAs) supply oxygen-rich blood (ORB) to the heart and consist of three layers: tunica adventitia, tunica media, and tunica intima [27]. The CAs divide into the left (LCA) and right (RCA) coronary arteries. The LCA give rise to the left anterior descending artery (LAD) and the circumflex artery (LCX). LAD and LCX delivers ORB to the left anterior and posterolateral side of the heart. While RCA and its branches (the right descending and marginal arteries) supplies ROB to the right side of the heart and nourishes the sinoatrial and atrioventricular nodes, which regulates the heart rhythm [28].

#### 2.1.2. Atherosclerosis

The term “atherosclerosis” was first introduced by Flix Marchand in 1904 [29]. In 1908, Alexander Ignatowski illustrated the relationship between a cholesterol-rich diet and experimental atherosclerosis [30]. The chemist Adolf’s work demonstrated that atherosclerotic lesions contain significantly more cholesterol compared to healthy arteries [31,32]. Nikolai Anichkov demonstrated that cholesterol could directly develop atherosclerosis in rabbits, thereby creating the first in vivo model [33]. In 1999, Russel Ross (1929–1999) introduced the response-to-injury hypothesis, linking endothelial damage to the onset of atherosclerosis, providing a deeper understanding of vascular disease [34,35].

**Figure 2:**
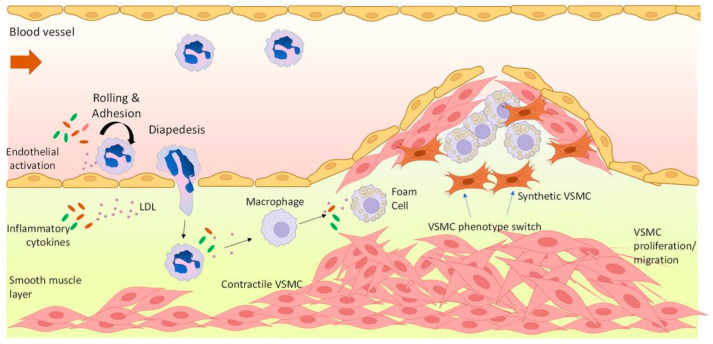
Schematic illustration of atherosclerosis initiation and progression over time [42].

Atherosclerosis is described as the hardening of coronary arteries caused by the build-up of cholesterol and fat particles in the inner wall, called atherosclerotic lesion. The mechanism by which atherosclerosis plaque starts to form, as shown in Figure 2, occurs within the intimal layer. Low-density lipoprotein (LDL) particles, termed bad cholesterol, accumulate at and then enter the injured intima [36]. Endothelial cells express adhesion molecules (chemoattractant cytokines, or chemokines) to recruit monocytes from the bloodstream. Chemokines enable monocytes to migrate into intima where they become mature and differentiate into macrophages. Subsequently, macrophages express scavenger receptors that allow them to engulf oxidised LDL particles and transform into foam cells [37]. T lymphocytes also migrate into the intima, where they regulate the activities of immune cells along with endothelial and smooth muscle cells. To respond to the injury, the secretion of growth factors and cytokines by platelets and other cells (e.g., platelet-derived growth factor, termed PDGF) switches VSMCs contractile phenotype to the proliferative and migratory phenotype [38,39]. Subsequently, VSMCs proliferate and migrate from the tunica media into the intima [40]. VSMCs aggregate at the intima forming a fibrous cap around the injury to keep the potency of the lumen. This fibrous cap is vulnerable to rupture and thereby to inducing acute cardiovascular events [41].

#### 2.1.3. In-Stent Restenosis

**Figure 3:**
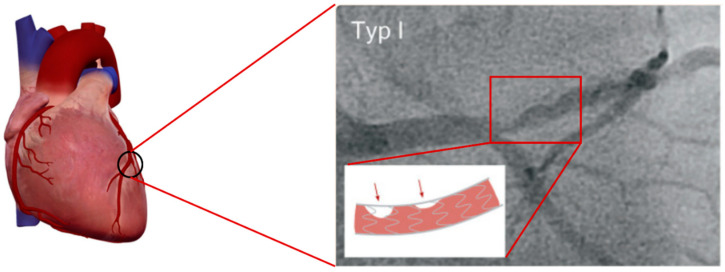
Coronary artery fluoroscopic image shows ISR during minimally invasive cardiovascular diagnostic procedure. A contrast agent is injected into the coronary artery via a 2 mm hollow catheter to visualise arterial structures. Source: [43].

In-stent restenosis, often termed ISR, refers to the gradual development of blockage within a previously deployed stent (Figure 3). This phenomenon typically manifests between 3 and 12 months after its implantation [43]. Treating narrowed (stenotic) blood vessels with a stent widens the vascular lumen, but this procedure can sometimes lead to adverse reactions. One such reaction is the damage to the endothelium layer, which may occur due to many factors including the stent type such as BMS and DES [44]. The stent type plays a key role in initiating a vascular inflammatory cascade. Consequently, stent-induced damage triggers the accumulation of fibroblasts and the development of VSMC neointimal hyperplasia, which is the primary pathway for in-stent restenosis that drives the re-narrowing of the vessel [45].

#### 2.1.4. Risk Factors

Indeed, atherosclerosis begins in early childhood as an accumulation of fatty streak in the intimal layer [46]. These fatty streaks may either remain stable, progress, or regress. The risk factors of CAD are various and thus divided into non-modifiable and modifiable [47]. Table 1 summarises factors leading to the development of atherosclerosis.

#### 2.1.5. Symptoms

The symptoms of CAD can sometimes be silent, until a heart attack occurs [60]. Common symptoms include chest pain (angina), shortness of breath, arm or shoulder pain, cold sweats, and weakness. Angina results from the narrowing of the coronary arteries, which restricts blood flow to the heart. It often manifests as a tightness in the chest that intensifies with physical activity and eases at rest [61]. A heart attack occurs when coronary arteries are severely narrowed or completely blocked, leading to irreversible heart damage and death if untreated [62]. CAD may also lead to heart failure (HF), where the heart cannot pump sufficient blood to meet the body’s demands. This leads to blood accumulation in the chest, leading to shortness of breath [63].

#### 2.1.6. Current Diagnosis

There are multiple methods to diagnose and confirm CAD. Diagnosing modalities include electrocardiogram (EKG), echocardiogram (echo), stress test, chest X-rays, blood test, and cardiac intervention [64]. EKG is a standard test that not only assesses electrical activity (heart rate and rhythm) of the heart but also enables the predictions of the quality of the heart muscle. For example, ST-elevation myocardial infarction, or STEMI, is a change in the electrical pattern shown on the EKG, indicating a condition of an ongoing acute coronary syndrome (heart attack) [65]. while Echocardiography is a non-invasive ultrasonic imaging technique of the heart, capable of providing valuable diagnostic information about cardiac haemodynamic and function. It can precisely evaluate the motion of the heart wall, chamber size, heart valves, pericardial cavity, and blood ejection fraction [66,67]. In addition, a chest X-ray is a painless and quick diagnostic tool of the heart. It is often among the first choices ordered by the physicians. Changes in the structure and size of the heart may indicate HF or valvular disease. Moreover, it can also reveal the presence of an aortic aneurysm or calcium deposits in the heart [68]. The stress test is a method of evaluating the effectiveness of the heart during physical activities. Patients will be asked to walk on treadmills or stationary bikes where health practitioners watch and record heart rhythm and blood pressure. This would guide healthcare providers to diagnose the heart and any abnormal rhythm [69]. Blood sample tests can then validate the diagnosis. For example, an excess of natriuretic peptides and troponin T proteins in the blood indicates a diagnosis of myocardial infarction [70]. Results from these test might indicate CAD and presence of the so-called “culprit lesion” within the coronary circulation, necessitating diagnostic cardiac intervention using PCI as necessary.

#### 2.1.7. Current Treatments

The development of atherosclerotic lesions within the coronary inner wall causes narrowing to the lumen, and thus, restricts the blood flow to distal parts of the heart. To alleviate the symptoms of CAD, patients can undergo either PCI, coronary artery bypass graft (CABG), or medications for minor conditions. PCI is a non-surgical procedure where a well-trained physician obtains vascular access at patients’ groin area (femoral artery) or at the arm wrist (radial artery). Through this vascular access, a flexible thin hollow tube is inserted and threaded up to the heart. The catheter tip is then engaged into the LCA or RCA ostia (the opening of the vessels). Materials such as balloons, stents, and medications are delivered through the catheter to the injured site. Commonly, PCIs start by the insertion of a small balloon and inflate it to squash fatty deposits against the CA wall and thereby widen the lumen. After ballooning the vessel, a stent is then delivered and inflated to keep the lumen open. Stents act like a scaffold and stay in the heart permanently.

CABG, also called open heart, is a surgical procedure to allow blood to reach ischemic tissues when PCI becomes not ideal. During CABG procedures, a surgeon uses healthy blood vessels from other body parts to bypass severely stenotic or clogged areas. This procedure is essentially recommended in individuals with severe stenosis or multiple coronary arteries. The left internal mammary artery (LIMA) from the chest and the saphenous vein from the leg are the most commonly used grafts. The LIMA, termed arterial graft, has a better survival rate, whereas the saphenous vein, termed vein graft, has poor outcomes in long-term follow-up [71,72].

In addition to lifestyle changes, treating coronary artery disease with medications to alleviate symptoms and lower the risk is possible. Generally, medications include antiplatelets, antihypertension, cholesterol-lowering, and antiarrhythmic drugs. See Table 2 that summaries the commonly used medications for CAD.

## 3. Chronic Kidney Disease

Healthy kidneys operate to filter the blood by removing toxic substances and play a crucial role in regulating blood pressure [76,77]. CKD is another intractable condition where the kidneys become damaged and unable to function properly. Clinically, CKD is defined as a structural abnormality of the kidneys, represented by a decreased glomerular filtration rate (GFR) of less than 60 mL/min/1.73 m^2^. End-stage kidney disease (ESKD), the most severe form of CKD, is characterised by a GFR of less than 15 mL/min/1.73 m^2^. Consequently, patients that suffer from ESKD must receive haemodialysis treatment or seek a kidney donor for transplantation [78,79].

The burden of CKD has been identified as a worldwide epidemic disease in the last three decades [80]. The impact of CKD reaches more than 800 million people, compromising roughly 10% of the global population [81]. By 2040, it is expected that CKD will be re-ranked from the 16th to the 5th leading cause of death [82]. The prevalence of CKD escalates with advancing age. In England, estimates suggest that the prevalence of CKD affects 15% of people aged 35 years and older. In the US, the prevalence of CKD is estimated at 14.8%, and thus, affects an enormous population of roughly 30 million adults [83]. The mortality rate has significantly increased in the last 10 years by one-third, accounting for 1.2 million deaths globally [84]. Indeed, individuals suffering from CKD are highly susceptible to sudden death due to specific factors including CVD or infections [85].

Diabetes and hypertension are common risk factors and are considered as the primary culprits behind the impairment of kidneys. Other risk factors include nephritis, cystic kidney disease, age, obesity, and a family history of CKD [86,87]. There are robust similarities in risk factors between CKD and CVD. These risk factors are explained in Table 2. Haemodialysis, an external artificial kidney, is an ideal treatment for ESKD. The process involves the simultaneous transportation of blood waste products such as urea to dialysate and bicarbonates from dialysate into the blood [88]. A patient should first undergo an operation to have arteriovenous access (AV) that facilitates the process of kidney replacement therapy. The most commonly used AV accesses are the AV fistula, AV graft, and venous central line [89]. Each access will be evaluated by itself in separate sub-headings.

### 3.1. AV Fistula, AVF

**Figure 4:**
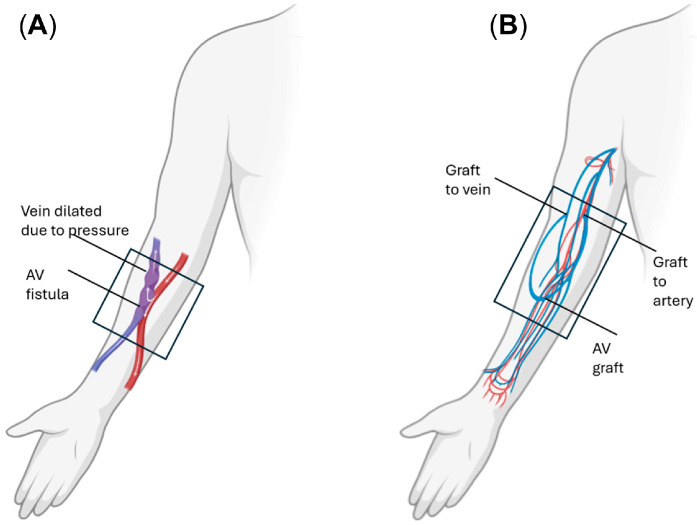
Schematic illustration of haemodialysis accesses. (**A**) Arteriovenous fistula; (**B**) arteriovenous graft [103].

AVF is a surgical procedure in which a high-pressure artery is connected to a low-pressure vein; see Figure 4A. The creation of AVF is common in ESKD patients to perform long-term haemodialysis [90]. This access is preferred by surgeons because it provides a high flow rate suitable for effective dialysis. Also, AVF has minimal complications and can be used multiple times a week for haemodialysis. Several studies supported the use of AVF access due to its low infection rate, hospitalisation, catheter failure, mortality, and its low cost compared to other modalities [91,92,93]. The primary drawback of AVF is its long period of maturation, which may take 4–6 weeks, with an approximate failure rate ranging from 30% to 50% [94]. This failure could be due to many factors such as age, race, gender, vascular disease, diabetes, and fistula location. Other complications of AVF non-maturation can arise from an early thrombosis formation [95].

### 3.2. AV Graft, AVG

In contrast, AVG, as depicted in Figure 4B, is a synthetic conduit used to connect an artery with a vein that lends itself to the incorporation of biosensors. Creating AVF access can sometimes be impossible to achieve due to previous veins damage from medical interventions. In such situations, the use of these prosthetic grafts can overcome this obstacle [96]. Fortunately, grafts allow frequent cannulation instead of the native blood vessels. These grafts are typically constructed from PTFE with different diameters ranging from 4 to 8 mm [97]. Understanding the anatomy of blood vessels is essential, as it will enable the proper handling of haemodialysis access. Based on the anatomical locations, grafts are anastomosed onto native vessels either in a straight or looped manner. Therefore, common configuration can be looped in the forearm or straight in the upper arm [98].

Even though chronic vascular access may accompany various complications including thrombosis, aneurysm, infection, or venous hypertension. These complications tend to appear more often with AVGs over fistulas [97,99,100]. One significant and common complication of AVGs is the occurrence of stenosis, especially at the graft–venous junction, called venous stenosis. And venous stenosis occurs due to neointimal hyperplasia and is the main cause of graft failure as well as thrombosis [101,102].

## 4. Biosensors in the Field of Healthcare

There is a pressing need for healthcare providers to enhance services and reduce costs. Integrating biosensors for disease detection and surveillance is one such solution for improved patient health management. Such technologies would assist in disease diagnosis, prevention, and enabling individualised treatment. A biosensor is a microdevice capable of sensing biological and chemical changes such as cells and tissues and translating acquired data into electrical signals [104]. Their use dates back to 1956, when the oxygen detection biosensor was developed by Leland Clark, “the father of biosensors”.

**Figure 5:**
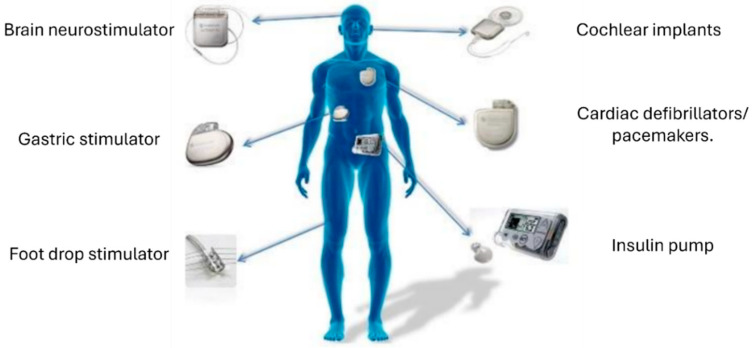
Applications of implantable biomedical devices. Source: [109].

Biosensors are widely employed in various applications including healthcare, as shown in Figure 5. For instance, continuous glucose monitoring devices offer diabetic patients a solution for tracking their glucose levels in the blood at all times, sharing outcomes, and sending alerts of incidences of low and high glucose levels [105]. In 2017, the US Food and Drug Administration (FDA) approved Abbott’s Freestyle Libre, a disposable glucose monitor that can be applied to the back of a patient’s arm for up to 14 days [106]. Similarly, cardiac electrocardiography rhythm monitoring systems, such as AliveCor’s “KardiaMobile”, enable ECG observation at home, offering patients a convenient solution for cardiac rhythm monitoring [107].

The rapid advancements in the field of biosensor technology are the result of extensive collaboration between scientists across various disciplines, including biology, engineering, bioelectronics, and nanotechnology. This interdisciplinarity is essential to revolutionising the field of healthcare, delivering high-quality and cost-effective health solutions. Currently, we notice that healthcare services are gradually shifting away from traditional centralised systems and entering the era of technology and digitalization, such as mobile health; this transformation will enormously support health decision-making [108].

### 4.1. Smart Implants for Cardiovascular Disease

In recent years, healthcare providers and researchers have shown increasing interest in telemedicine to monitor heart rhythms and mitigate adverse reactions associated with CVD and PVD therapies. For example, observing abnormal heart rhythms and monitoring ISR post-treatment. They believe that the coronavirus pandemic (COVID-19) has strongly encouraged a shift from providing “in-office” health service to remote monitoring. They assume that remote monitoring is a key to improving healthcare systems by increasing the accessibility of more people to health services and offering personalised treatment [110].

The innovation and development of smart implantable devices for CVD is not an entirelynew endeavour; as, some devices have already been commercialised with, others in development. For example, CardioMEMS^TM^ is a small implantable device (15 × 3.4 × 2 mm), see Table 3, developed by the Abbott company used to remotely monitor the pulmonary artery pressure of patients with HF [111]. CardioMEMS^TM^ consists of three main components: (1) inductor, (2) pressure-sensitive capacitor, and (3) two nitinol loops. The capacitor is made of two conductive layers separated by a space medium. When pressure is applied, these conductive layers move towards each other, decreasing the space between them and altering the capacitance of the circuit. This change causes a shift in the resonant frequency of the circuit, which is directly correlated to the force exerted on the sensor’s surface. Both inductor and capacitor are encapsulated within medical-grade biocompatible silicone. Indeed, biocompatibility and adherence of ISO standard 10993:2018 are an essential element of the regulatory process (See https://www.fda.gov/, accessed on 9 September 2024). CardioMEMS^TM^ relies on wireless electromagnetic coupling technology to share data with cardiologists from the patient’s home. Such technology enables HF management, reduces frequent inpatient admissions, and potentially reduces the cost [112,113].

In addition, an implantable loop recorder, such as the Medtronic’s Reveal LINQ^TM^ (Table 3), is a heart rhythm monitor. This device can be implanted under the skin in the chest for up to 3 years, where it records any abnormal activity of the heart’s electrical rhythm [114]. The size of this device is 7 × 45 × 4 mm, and it is equipped with wireless telemetry to transmit data (recorded ECG) to the clinic, wirelessly [115]. For security and privacy, Reveal LINQ^TM^ transmits data daily to an external unit, called MyCareLink^TM^, monitoring within a range of up to 6 feet. Then, MyCareLink^TM^ requires a 3G network connection for wireless data transmission [116].

**Figure 6:**
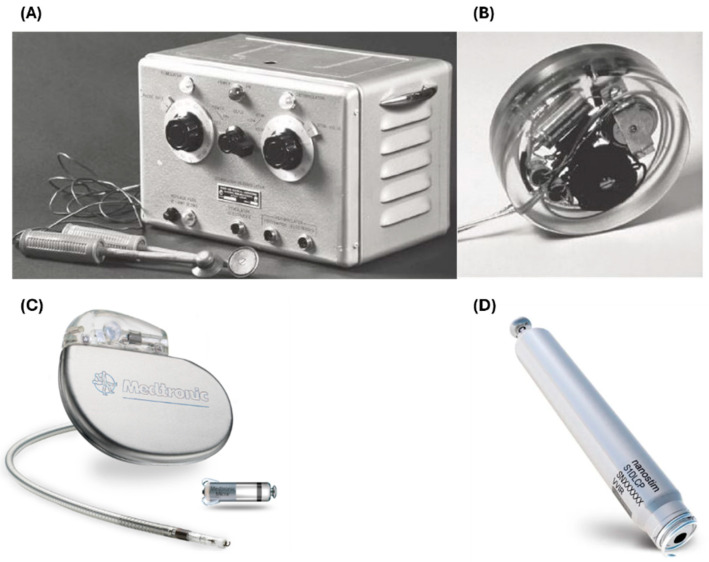
Pacemaker development over time. (**A**) shows the first invented non-implantable pacemaker by the Canadian biomedical engineer John Hopps; (**B**) shows the first implantable pacemaker implanted by cardiac surgeon Ake Senning; (**C**) illustrates the Medtronic implantable pacemaker, in addition to the leadless intracardiac Micra^TM^, which is more than 90% smaller than typical pacemakers; (**D**) represents the intracardiac Nanostim^TM^ pacemaker by Abbott (formerly St. Jude Medical) [117].

The treatment of abnormal heart rhythms started in the 20th century when John Hopps, a Canadian biomedical scientist, designed and built the first non-implantable pacemaker (Figure 6A). His pioneering work led to the development of the first implantable pacemaker by Rune Elmqvist (Figure 6B). In 1958, the first implantable pacemaker was entirely inserted in a patient in Stockholm, Sweden, by cardiologist Ake Senning [117,118]. In early 1970, Dr. Spickler introduced the idea of producing leadless pacemakers to encounter the challenges of traditional pacemakers such as lead dislodgment and short battery life [119,120]. After a few decades, St. Jude Medical Inc. (now known as Abbott) developed Nanostim^TM^, the first leadless pacemaker that was completely implanted in the right ventricle of a human’s heart. Nanostim^TM^ received the CE mark in 2013 and become the first intracardiac pacemaker to be launched across Europe. Similarly, Medtronic developed their first generation of a leadless pacemaker, called Micra^TM^ (Figure 6C), which is 93% smaller than their conventional pacemaker. Their breakthrough pacemaker received FDA approval by 2016. Importantly, these two types of pacemakers can function wirelessly [121,122]. Currently, leadless pacemakers rely on batteries with limited lifetime. On average, the Nanostim^TM^ (Figure 6D) can last up to 15 years, while the Micra^TM^ has a lifespan extend to approximately 12.5 years [123]. The limited lifespan and size of batteries has always been a limitation of such devices and makes their inclusion in smart stents impractical, whereas their use is more feasibly in smart grafts or with the use of wireless power [124].

The heart consists of four chambers: two atria that receive blood from the body and two ventricles that pump blood out. These chambers have unique pressures that differ from one another based on the chamber’s function. For example, the systolic pressure in the left ventricle and right ventricle is roughly 120 and 20 mmHg, respectively. This is because the left ventricle has to pump blood throughout the entire systemic blood circuit. Oppositely, the right ventricle pumps blood only throughout the pulmonary blood circuit (short distance) [125]. Evaluating the left atrial pressure (LAP) is important in patients with HF, congestive heart disease, and mitral valve disease. To measure intracardiac pressure, a special catheter, called Swan Ganz, is inserted into the femoral vein and threaded up to the right atrium, right ventricle, and pulmonary artery to record the pressure in each chamber. This conventional method does not enable the real measurement of the left atrium and ventricle but rather provides an estimate [126]. A new wireless technology was created by Victorious, named V-LAP (Table 3), for monitoring LAP remotely. The V-LAP device can penetrate and stay in the interatrial septal wall. Such a device is capable of communicating with the external world at rest and ambulation [127].

### 4.2. Smart Implantable Devices for Non-CVD Diseases

**Figure 7:**
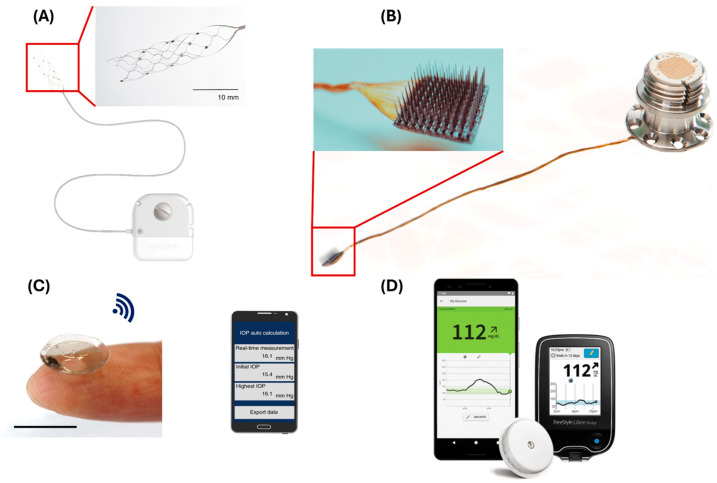
Applications of smart biomedical devices. (**A**) demonstrates one form of BCI, termed Stentrode^TM^ by Synchron. It consists of electrodes built onto the stent’s struts, capable of sensing from within the brain (Panel A is sourced from Synchron). (**B**) BCI NeuroPort^TM^, which consists of 96 electrodes developed by Blackrock (from Blackrock website). (**C**) shows smart eye contact lenses for the continuous monitoring of intraocular pressure developed by a Korean research team [134]. (**D**) shows Freestyle Libre for monitoring the glucose level in the blood. It enables continuous observation and displays results in a smartphone app [132].

Implantable biosensors will have a multiplicity of roles in the human body. Their applications expand to address diseases such as neurological, ocular, and metabolic (glucose level) diseases to name a few applications. Smart medical devices such as Stentrode^TM^ by Synchron (Figure 7A) is a stent with electrodes that can be inserted and deployed inside blood vessels in the brain. One such benefit of Stentrode^TM^ is that it can record signals from deep sites in the brain and parse these to effector devices such as human computer interfaces (BCI). This technology allows patients to control devices using their mental thoughts via wireless technology; for example, digital typing [128]. In particular, the electrodes on the Stentrode^TM^ sense the neurological activity and transmit signals through connecting leads to a processing and communication unit implanted in the chest. These signals are then decoded and sent to an external receiver to execute specific action. Recorded data are transmitted via Bluetooth wireless technology [129,130]. NeuroPort arrays are another form of BCI built by Blackrock Neurotech (Figure 7B) a start-up company. There device, consisting of 96 electrodes. The implantation of NeuroPort arrays on the brain enables sensing and recording neurons activity and thereby sends signals via connecting wire to an external device. These signals are then translated into commands and control objects such as a wheelchair or robotic hands [131,132]. Using Bluetooth technology for data transmission in deep-body implants is not practical [133]. Thus, implementing such technology in smart stents and grafts is not feasible.

The management of glaucoma requires the continuous monitoring of intraocular pressure. Korean researchers have been able to create smart contact lenses with an embedded sensor for the real-time monitoring of intraocular pressure (Figure 7C), and the wireless transmission of data via magnetic coupling to a smartphone app. Successfully, their approach showed promising results in both the animal model (rabbit) and the human model [134]. Furthermore, the integration of biosensors for the continuous monitoring of the blood glucose (Figure 7D) level introduced a paradigm shift in the management of diabetes. There are various commercially available devices include “Dexcom” and “Freestyle Libre”. Both devices transmit data wirelessly via Bluetooth to smartphone app [132].

**Table 3:** List of CVD and PVD smart implantable devices.

Developer	Abbott	Medtronic	Synchron	Medtronic	Vectorious
Device Type	Implantable sensor.	Subcutaneous implantable senor.	Neuro-stent prosthesis.	Implantable leadless pacemaker.	Intracardiac sensor and external belt.
Product	CardioMEMS. Commercially available.	Reveal LINQ. Commercially available.	Stentrode. Under development.	Micra^TM.^ Commercially available.	V-LAP
Indications	HF.	Cardiac arrhythmias.	Neurological disorders.	Cardiac arrhythmias.	HF. Mitral valve regurgitation. Atrial arrhythmias.
Function	Real-time monitoring of pulmonary artery pressure.	Observe heart rhythm continuously.	Stimulation of brain neurons and allows controlling external devices by thoughts.	Sense and record abnormal heart rhythm, and apply electrical pulse to stop unwanted activity in the heart.	Monitor left atrial pressure and send data to secure cloud. Clinicians can access data and adjust treatment accordingly.
Advantages	Managing HF. Offer treatment adjustment-based reading. Reduce hospitalisation. Reduce risk of acute decompensation. Safe implant. No battery. Wireless communication.	Easy implantation. Wireless data transfer. Data transfer up to 2 m High data security.	No open brain surgery needed. Minimally invasive procedure. Wireless transmission.	93% smaller than old pacemakers. Deep tissue wireless transmission. Light weight, 2 g. Long-term success. Corrosion resistant. Can be retrieved.	Enable direct measurement of left atrium. Not only for pressure. It can also detect other cardiac issues. Needs minimally invasive procedure. Secure wireless data transmission. Safe and accurate.
Disadvantages	High cost. Relies on bulky external device for data transfer. Limited to pressure monitoring.	Battery life (up to 3 years). Subcutaneous implant. Requires massive external unit.	Requires telemetry electronics in the chest.	Battery life (4.7 yrs).	Needs trans-septal procedure. Raises the risk of thrombosis.
Clinical trials	CHAMPION trail. COAST trail.	N/A	SWITCH trail NCT03834857.	The Micra Transcathet-er Pacing Study (TPS) [135].	NCT03775161, first in-human clinical study. Multicentre. Open-label clinical trial. Assessment of safety, usability and performance.
Biocompatibility (BC)	BC. Encapsulated in medical-grade silicone.	BC. Titanium casing.	NA	BC. Titanium casing.	BC.
References	[111,136,137,138].	[115,116,139].	[128,129,130,140].	[121,122,141].	[127].

### 4.3. Wireless Power Transfer to Medical Implants

One of the greatest challenges facing medical implants is the transmission of power wirelessly. If an efficient and safe solution of wireless power transfer (WPT) is achieved, medical smart implants will enter a new revolutionary era. WPT is defined as a wireless transfer of energy from an external source to power or charge the implantable device without connecting wires. Currently, there have been various attempts to deliver wireless power to implanted devices based on the principle of electromagnetic fields. Yet, difficulties in the miniaturisation of the antenna and its orientation have been an unresolved obstacle. Attempts to address these problems are divided into sections termed near-field, mid-field, and far-field WPT [142].

#### 4.3.1. Near-Field WPT

**Figure 8:**
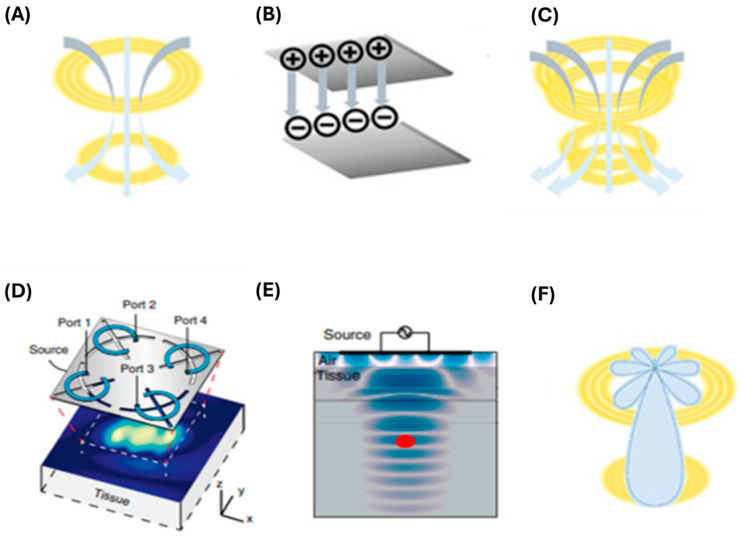
Schematic illustration of different methods of WPT. (**A**) IC between two short-distanced coils. The concept of IC was primarily invented by Nikola Tesla in the 19th century. (**B**) Capacitive coupling demonstrated by two plates acting as transmitter and receiver coils. (**C**) MRC contains four coils. (**D**) The innovative MF benefited from a patterned metal plate with four ports that focus energy on the targeted surface, enabling efficient power penetration through the tissue. (**E**) Theoretical illustration of MD energy propagation through the body tissue, reaching the biomedical implant (red dot) at a depth of >5 cm. (**F**) FF scheme showing radiation of RF in many lobes, but predominantly towards the receiving coil. Source: [143,149].

Near-field (NF) WPT is the most popular form of energy transfer for short distances, ranging from millimetres (mm) to centimetres (cm). NF-WPT can be divided based on the working principle into subcategories: inductive coupling (NFIC) (Figure 8A), capacitive coupling (NFCC) (Figure 8B), and magnetic resonant coupling (NFMRC) (Figure 8C). NFIC has been widely used to supply wireless power to various devices. For example, medical wearable devices, smartphones, and electric toothbrushes. Highly efficient power transfer for short distances and the simple implementation of two coils for transmitting and receiving power are the most common advantages of NFIC. In contrast, NFIC mandates precise alignment and short distance WPT that limits their use in medical deep tissue implantations [143]. To overcome these drawbacks, a new concept was proposed by Kurs et altermed NFMRC, in 2007. NFMRC technology exhibits a longer distance of energy transmission, up to a few metres, when compared to inductive coupling. Typically, the system consists of four coils: source coil, transmitter coil, receiver coil, and load coil [143,144]. Despite its advantages, like higher frequency bandwidth, optimal impedance matching, and a high Q-factor, its structural complexity poses a problem due to the requirement of multiple coils. Thereby, it becomes unsuitable for bio-implants [145]. Ultimately, NFCC is a WPT scheme that relies on an electrical field to deliver power. The system requires at least two parallel plates separated by a medium to form a coupling capacitor. These plates act as transmitter and receiver coils. Although this scheme is less sensitive to electromagnetic interference and the misalignment of plates, it is hindered by a greater bulk and low power output. An in vitro comparison between conductive and capacitive WPT by Aldaoud et al. showed that NFIC offers higher power transfer efficiency than NFCC [146,147] that could be suitable for powering future vascular implants.

#### 4.3.2. Mid-Field WPT

Currently, the inductive coupling technique is not suitable for powering implantable medical devices. Therefore, mid-field (MF) WPT (Figure 8D,E), has been introduced by Ada Poon et al., presents as an innovative solution capable of delivering efficient WPT to millimetre-sized deep tissue implants [148]. MF uses a specially patterned metal plate (dimensions 6 × 6 cm) with four slots to create an ideal field pattern. The metal plate is located in the air in close proximity to the skin. It serves as a cornerstone of the MF technique by inducing intensive and adaptive energy transmission through propagating modes in tissues. In particular, four independent radiofrequency (RF) ports send exciting signals through the structural slots on the metal plate, which manage the targeted surface of the field, thereby enhancing efficiency. With this innovative approach, milliwatt power can be transferred to micro-implants greater than five centimetres (>5 cm) in depth. Body tissue safety is importantly considered where MF continues to offer a lower risk of overheating and damage to surrounding tissues [143,149,150]. Consequently, the MF approach is currently the most preferred form of WPT for deep-body implants.

#### 4.3.3. Far-Field WPT

Far-field (FF) WPT (Figure 8F) is a method of power transmission over long distances, typically through RF electromagnetic waves or microwaves [151]. In FF-WPT, radiation is the key method of energy transmission from a transmitter to a further away receiver, often within the range of metres. However, the attenuation of human body tissues offers a significant obstacle to the adoption of this technology. On the contrary, NF-WPT continues to offer low efficiency in power transmission as the distance between transmitter and receiver coils increases, while FF-WPT enables the transmission of power in the form of radiation over distances within several metres. Nonetheless, FF-WPT efficiency is limited by the fact that transmission over long distances results in a loss of power density due to the dissipating electromagnetic field in space [152]. In addition, body tissues and fluids cause attenuation to electromagnetic waves, and thereby, this reduces the power received by an implantable device. For tissue safety, the Federal Communication Commission (FCC) imposes rules on how much power can be absorbed by the body. These exposure measures emerged to protect biological tissues from any potential damage that may be caused due to the overheating of powering an implantable device [153].

### 4.4. Wireless Communication and Data Transfer

Wireless technology is useful for delivering power to implantable devices but must adhere to regulated Specific Absorption Rates (SARs). That is, the power absorbed per mass of tissue in Watts/kg such as for use of implants during MRI scanning is covered under ISO/TS 10974:2018 (see https://www.fda.gov/). With transmitting recorded biological data to external units, WPT holds the potential to enable miniaturised and longer-lasting bio-systems compared to battery-powered alternatives. The previously mentioned WPT methods for transmitting power to biomedical implants can also be employed for data transmission, using so-called dual function antennas (DFAs). Near-field communication (NFC), a type of radiofrequency identification (RFID), is dominantly used for wireless data transfer within short distances. NFC offers secure wireless data transfer, with lower power loss and higher safety with human tissues. Thus, it is employed in many sectors, including medicine and businesses [143,154]. The communication between an implanted device and an external system can be divided into two sections based on the signal direction. Uplink refers to data that are sent from the implant to the external system, whereas downlink is used for signals sent from the external unit to the implanted device [155]. In general, two independent wireless links are thus far mandatory: one for power transfer and another for data transmission. Power transfer is more effective with narrowband links at low frequencies, as they show high power transfer efficiency and are less susceptible to noise and dissipation in the body tissue. In contrast, data transmission demands wider bandwidth links that operate at higher frequencies to leverage high-rate data transfer [156]. Having two separate links introduce multiple coils is known as a major drawback. This is because it increases the complexity and bulkiness of the biomedical device in addition to introducing unwanted signal interference. Conversely, a single pair of coils has been proposed to carry power and data simultaneously over a single frequency. The single-frequency principle has fundamental benefits that help reduce device complexity, size, and cost. However, the use of a single wireless inductive link to achieve simultaneous high power efficiency and high data rates is the desired goal but remains challenging. However, it is expected to overcome this challenge by using appropriate modulation techniques for power and data transmission over a single link [157].

Modulation techniques are defined as a conversion of signal waves into carrier waves to transmit data. This occurs by applying changes to the signal wave parameters such as amplitude, phase, and frequency. Using modulation techniques introduces numerous advantages, including low power consumption, less sensitivity to noise, and high-rate data transmission [158]. Currently, several modulations have been used to wirelessly transfer data from external units into implants (downlink). These modulations are amplitude-shift keying (ASK), phase-shift keying (PSK), and frequency-shift keying (FSK). In contrast, the load-shift keying (LSK) technique is particularly used for data transmission from implants to external units (uplink). Each of the aforesaid modulations is hindered by constraints. For instance, the ASK technique can be affected by nearby noise, increasing the bit error rate, while LSK suffers from a significant low transfer of power and data due to mistuning between the on/off states [143,159].

### 4.5. Future Direction—State-of-the-Art for Cardiovascular Smart Stents/Grafts

NIH and thrombosis continue to be primary threats to the failure of CVD and PVD stents and grafts. Restenosis commonly occurs in nearly 10% of patients with DES and as high as 50% in vascular grafts [81,160]. Restenosis, which can sometimes be silent and asymptomatic, is associated with irreversible health conditions such as morbidity and mortality. Often these complications mandate the patient to be readmitted to the hospital for a second operation. For example, undergoing another PCI procedure or re-establishing AV access. Repeated procedures afflict patients and cause further deterioration to their physical and mental health. Furthermore, they increase the burden on the healthcare system in multiple aspects. This includes the strain on medical staff and the substantial spending to correct complications that arise from current therapeutic solutions. Therefore, the transition to implantable smart devices has become essential for enhancing health and improving societal well-being. The employment of miniaturised implantable bioelectronics, biosensors, and wireless technology in CVD and PVD will permit the early detection of the onset of the disease and enable wireless pre-emptive treatment at an early stage. Early diagnosis and remote treatment would entirely reshape the healthcare system. Yet, the development of smart stents and grafts for early diagnosis and therapy is still ongoing. Some solutions have been achieved, but they are limited by drawbacks that prevent their use in humans.

**Figure 9:**
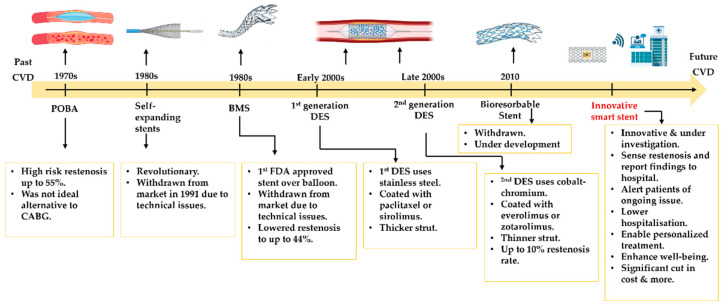
Illustrative diagram showing CAD evolvment over years. It is noted that the 2nd DES lowered the incidance rate of restenosis, but still pose threat to blood vessel. Innovative smart stents/grafts are thought to be able to address these challenges and lower their the risk of adverse reaction through wireless proactive teatment. This figure created by Biorender.

There have been several attempts to integrate and incorporate sensors into cardiovascular implants to overcome challenges facing both patients and healthcare providers (Figure 9). Specifically, the miniaturisation of microelectronics must be overcome. Here, we review some of these studies. Musick et al. developed a self-reporting stent to monitor the re-endothelialisation process using piezoelectric microcantilevers. These were integrated at multiple locations on the surface of a stent. Musick et al. hypothesised that the incomplete coverage of stent struts by endothelial cells leads to late thrombosis [161]. The real-time monitoring of this healing process would allow physicians to personalise anti-platelet prescriptions based on patient needs [162]. However, the technology’s inability to differentiate between various cell types or circulating substances, such as fibrin, clots, or endothelial cells, made it impractical.

On the other hand, Takahata et al. and his team developed an antenna stent by integrating a Micro-Electro-Mechanical System (MEMS) capacitive pressure sensor at one end. Combining pressure sensors with the stent forms an inductor–capacitor tank, which yields a resonant frequency, which reflects the blood pressure within the stent. Fluctuations in blood pressures indicate that ISR has occurred [163]. Furthermore, they adopted the commercially available double-J ureteral stent to enable the continuous monitoring of renal hypertension (hydronephrosis). This innovative approach is achieved by incorporating an inductive antenna with an integrated capacitive micro-pressure sensor to the wall of a ureteral stent [164,165]. Both of their approaches for CVD and CKD smart stents are effective for only the early detection of the onset of restenosis and cannot support wireless treatment. Therefore, this technology does not protect patients from illness, requires hospital visits, and leaves the healthcare cost burden unresolved. In addition, another unique design of a smart stent, called the helix-like stent antenna, has been developed by Liu et al. to serve as a whole stent antenna with two sensors integrated at both ends and one radiofrequency microprocess at one end. Pressure sensors at both ends measure the blood pressure and flow and transfer acquired data from the internal radiofrequency integrated circuit and antenna stent to an external antenna [166].

**Figure 10:**
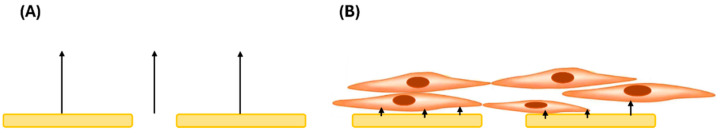
Schematic illustration of the concept of electrical impedance spectroscopy. (**A**) shows the current passing through a conductive substance such as cultural media. (**B**) shows cells proliferation and settlement over gold electrodes, deposited on the surface of a glass slide, where the current is impinged by cellular membrane.

Alternatively, electrical impedance spectroscopy (EIS), depicted in Figure 10, is a unique technique for an in vitro real-time monitoring of cell behaviour. The concept of studying cell proliferation and growth using the EIS technique was first utilised by the pioneers Giaever and Keese in the 1980s. When cells spread and settle over the sensing electrodes, they impinge the flow of alternating current, changing the impedance value. The change in impedance delivers essential information about the cell type, growth, and movement [167,168].

**Figure 11:**
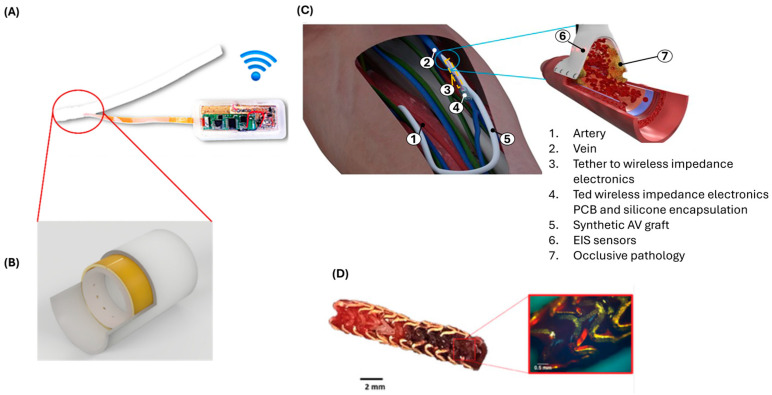
Smart graft/stent prototypes. (**A**) An assembled smart graft that consists of three main components: 1. A commercially available PTFE graft. 2. A 16-electrodes biosensor. 3. Implantable electronics encapsulated in biocompatible silicone. (**B**) An illustration showing the mechanism by which the polyimide sensor was wrapped around the wall of the graft, forming a circumferential ring shape. (**C**) A schematic illustration of smart graft connection between an artery and a vein, where the sensor is linked to the vein. (**D**) A depiction of a smart stent within which a blood clot was formed and detected. Source: [81,170].

Connolly et al. developed a smart stent specifically designed for ISR detection, following EIS technology. In their design, the stent functions as one electrode and a silver–silver chloride wire as another electrode [169]. Currently, Mercer et al. and his team at the University of Glasgow have exploited the EIS approach to develop the first self-reporting smart graft/stent, illustrated in Figure 11, for the early detection and prevention of vascular disease, such as ISR and thrombosis. For the smart graft, they integrated a 16-electrode polyimide sensor into the wall of PTFE and connected it to micro-implantable electronics. In a porcine model (in vivo), one end of the graft with electrodes was attached to the venous system, while the other end was linked to the arterial system. A small pocket was created in the neck to accommodate the electronics. Their translational work showed that it is possible to wirelessly detect and treat vascular disease. In addition to the smart graft project, Mercer’s group developed a smart stent, where the entire stent’s structure functions as a sensor. This is achieved by converting the stent’s strut into two distinct positive and negative electrodes, separated by non-conductive segments. This design allows the detection of restenosis over a larger area, rather than being limited to areas that are covered by discrete electrodes [81,170].

## 5. Conclusions

CVD and PVD continue to be the leading causes of death around the world, surpassing all forms of cancer and Alzheimer’s diseases combined. The occurrence of ISR in coronary artery patients and AV access closure in chronic kidney patients often leads to frustration for both patients and clinical providers. A narrowed or blocked coronary artery requires a stent to support its patency, but during the process of stenting, mechanical damage may arise and cause injury to the endothelial cell layer. Losing the endothelium integrity triggers an atherosclerotic inflammatory cascade leading to neointimal hyperplasia. Subsequently, a build-up of cells causes the re-blocking of that stented segment, and this is called ISR.

Similarly, CKD patients require permanent AV access to facilitate the process of haemodialysis. Commonly used accesses include AVFs and AVGs, each with their own benefits and drawbacks. These two forms are prone to closure and failure. AVFs is the most preferred form due to its low infection rate, but it needs up to 6 weeks to mature and become ready to be used. This maturation period adds an extra burden on patients who need immediate haemodialysis, whereas AVGs offer immediate haemodialysis and can also be cannulated multiple times. AVGs have a risk of becoming useless due to neointimal hyperplasia development, especially at the connection between the vein and the graft.

Converting the conventional stents and grafts into smart versions by integrating biosensors will transform the field of cardiovascular medicine. Biosensors that are capable of remotely monitoring the intravascular environment, sending early pathological warning signs, and even allowing wireless therapy will be transformational.

The innovative approach described in this review holds the potential to stratify patient needs and enhance the patient’s experience. In parallel it will improve outcomes, and alleviate the economic and healthcare burden on global healthcare budgets.

## Figures and Tables

**Table 1:** Risk factors of atherosclerosis.

	Risk Factors	Comments
Non-modifiable	Age	Age is associated with a high risk of atherosclerosis in people greater than 65 years old. The functions of mitochondria decrease, and Interlukin-6 increases with ageing; however, these effects accelerate the process of atherosclerosis [48].
	Sex	Women are less likely to develop CAD, especially perimenopause [49]. This fact may be due to the oestrogen protection of young females [50].
	Race	In the US, Black, Hispanic, Asian, and American Indian people are at a higher risk of developing CAD than American whites. This is due to wealth, quality of healthcare and education, safe environment, and community for white people [51].
	Familial history	“The multi-ethnic study of atherosclerosis” linked familial history to the development of atherosclerosis [52].
Modifiable	Hypertension (HT)	Epidemiological studies stated that arterial HT accounted for 18% of CAD and 48% of stroke events [53].
	Diabetes (Type-II)	Atherosclerosis occurs earlier in diabetic than non-diabetic populations. In total, 75% of diabetic patients die due to complications of CAD [54,55].
	Smoking cigarettes	There is evidence that smoking can exacerbate CAD severity [56].
	Obesity	Obesity is an independent risk factor that contributes to CAD. Epidemiologic studies showed that there is a strong association between CAD and obesity [57].
	Hyperlipidaemia (ex, LDL)	Excessive LDLs in the blood enable their adherence to the arterial wall, followed by atherosclerosis [58].
	Lifestyle	Lifestyle behaviours are the main cause behind the development of other risk factors. For example, unhealthy diets lead to type II diabetes, obesity, hypertension, or high cholesterol (LDL) [59].

**Table 2:** Commonly used medications for the treatment of CAD.

Medications	Medicines	Comments
Antiplatelets	Acetylsalicylic acid (ASA)	A low dose of ASA is recommended as a blood thinner and to lower the risk of CAD events. Patients with CVD history are advised to use it lifelong.
	Clopidogrel	It prevents platelets from bonding together, especially in patients who are at a high risk of CAD.
Cholesterol-lowering drugs	Statins	Statins are widely used to block the pathway by which the liver produces cholesterol via the inhibition of HMG-CoA reductase. (Hydroxymethylglutaryl-coenzyme A)
	Ezetimibe	It inhibits cholesterol absorption in the intestine and biliary tract. This helps reduce LDL cholesterol significantly. Ezetimibe can be prescribed in combination with statins [73].
Antihypertension: beta blockers	Acebutolol, bisoprolol, and metoprolol	Beta blockers inhibit adrenaline and noradrenaline which result in slowing the heart rate and reducing blood pressure. These drugs are convenient for hypertensive patients with CAD as they relieve chest pain (angina).
Antihypertension: calcium channel blockers or CCB	Amlodipine, diltiazem	CCB causes the heart to relax via blocking cell membrane calcium gates and thus preventing calcium from entering cardiac cells. This pathway causes blood vessels to widen and lower the blood pressure.
Antihypertension: ACE inhibitor	Benazepril, captopril, and enalapril.	ACE inhibitors stop the conversion of angiotensin I into angiotensin II by blocking the angiotensin-converting enzyme. This action reduces the blood pressure and additionally prevents VSMCs’ remodelling process [74].
Antihypertension: angiotensin II receptor blockers (ARBs)	Valsartan	ARBs are recommended for those with ACE inhibitor intolerance. They bind to angiotensin II type I receptors and inhibit the angiotensin II effect on blood pressure. This drug class is used to manage blood pressure and diabetic nephropathy [75].

## Data Availability

Data upon request to corresponding author.
